# Triple Threat: Triple Pathogen Endocarditis

**DOI:** 10.7759/cureus.47860

**Published:** 2023-10-28

**Authors:** Jessica M Gonzalez, Gabriel Lowenhaar, Moti Ramgopal, Prasad Chalasani

**Affiliations:** 1 Department of Internal Medicine, Brown University, Providence, USA; 2 Department of Emergency Medicine, Brown University, Providence, USA; 3 Department of Infectious Disease, Florida State University College of Medicine, Fort Pierce, USA; 4 Department of Cardiology, Florida State University College of Medicine, Fort Pierce, USA

**Keywords:** infective endocarditis, pseudomonas aeruginosa (p. aeruginosa), gemella haemolysans, streptococcus anginosus group, polymicrobial endocarditis

## Abstract

Polymicrobial endocarditis is rare but is seen in those with risk factors like diabetes mellitus, structural heart disease, congenital heart defects, prosthetic devices, and intravenous drug use. We report the case of a 30-year-old woman with a past medical history of chronic Hepatitis C and IV drug use who presented with a one-week history of generalized weakness, subjective fevers, lower extremity abscesses, and occasional chest pain. Blood cultures were positive for* Streptococcus anginosus*, *Gemella hemolysans*, and *Pseudomonas aeruginosa*. A transthoracic echocardiogram revealed a very large tricuspid valve vegetation and severe tricuspid regurgitation. Her course was complicated by a complete heart block, septic pulmonary emboli, acute hypoxic respiratory failure, and cardiogenic shock meeting the criteria for early surgical intervention. She underwent an emergency tricuspid valve replacement and pacemaker implantation. During the operation, it became evident that her valve was destroyed with vegetation. A week after the operation, her ejection fraction had improved to 50% and she only exhibited mild tricuspid valve regurgitation. Six weeks later, she was in a stable condition and presented for follow-up. Surgery is necessitated in nearly 50% of *Gemella *endocarditis cases, 62% of cases with *S. anginosus* group, and approximately 56% of *P. aeruginosa* cases. To our knowledge, this is the only case of polymicrobial endocarditis caused by *G. hemolysans, S. anginosus,* and *P. aeruginosa.*

## Introduction

There has been an increase in the incidence of hospitalizations due to infective endocarditis (IE) [1]. IE has variable clinical manifestations and high mortality [2-5]. Common pathogens causing IE include *Staphylococcus aureus*, viridans streptococci, enterococci, and coagulase-negative staphylococci [1]. Infections caused by additional or multiple organisms (i.e., *Pseudomonas* and *Gemella*) are rare [2-5]. Diagnostic tools include blood cultures and echocardiography; blood cultures allow us to determine medical treatment options while echocardiography provides us information on the size of vegetation, valve function, and ejection fraction which can determine the need for surgical intervention.

## Case presentation

A 30-year-old woman with a past medical history (PMH) of chronic hepatitis C and IV drug use presented with generalized weakness, subjective fevers, lower extremity abscesses, and occasional chest pain. Her labs on presentation revealed severe anemia, thrombocytopenia, and hyponatremia (hemoglobin was 7.1, platelet count was 68, and sodium was 130). A chest x-ray demonstrated cardiomegaly, central congestion, bilateral pleural effusions, and infiltrates suggestive of septic pulmonary emboli. She was started on vancomycin and piperacillin/tazobactam.

**Figure 1:**
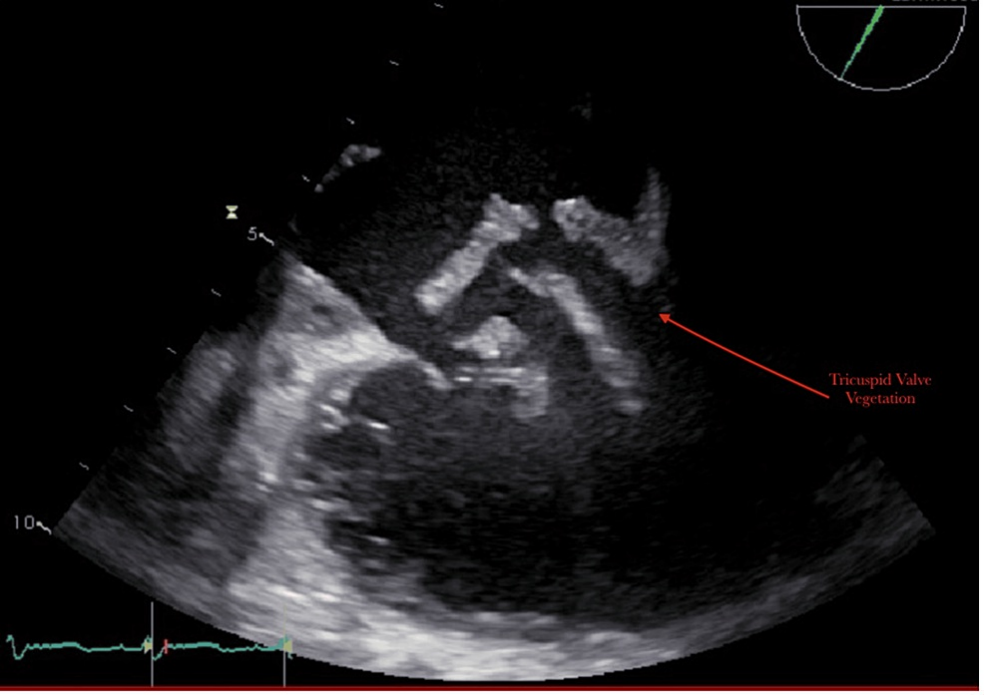
Large vegetation on tricuspid valve

**Figure 2:**
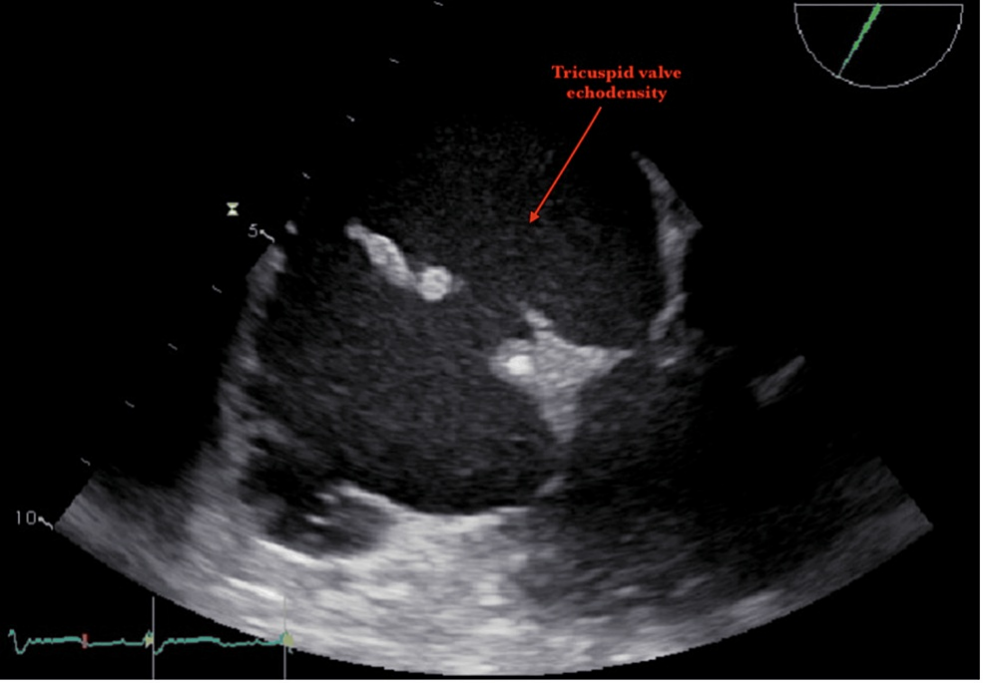
Tricuspid valve floating vegetation

Blood cultures were positive for *Pseudomonas aeruginosa* and *G. hemolysans*. A transthoracic echocardiogram revealed that the left ventricle was moderately dilated, and the left ventricular ejection fraction (LVEF) was 30%. There was a very large tricuspid valve vegetation (approximately 20mm) and severe tricuspid regurgitation (Figures 1, 2). At first, she was hesitant about surgery and opted for medical management. On her second day of hospitalization, she developed worsening acute hypoxic respiratory failure requiring chest tube placement. After four days of antibiotics, her blood cultures continued to grow *P. aeruginosa* and *G. hemolysans. *Her hospital course was also complicated by a third-degree AV block (complete heart block) on her fifth day of hospitalization. She ultimately elected to pursue surgery after five days of medical management. She was taken to the operating room for an urgent tricuspid valve replacement with a Carpentier-Edwards Magna ease valve and the insertion of a permanent RV epicardial lead. During the operation, it was revealed that the valve was completely destroyed with vegetation. Intraoperative cultures of the vegetation were positive for *P. aeruginosa* and *Streptococcus anginosus *(Table 1). She had an uncomplicated postoperative course. She met with the addiction medicine team during her hospitalization and was scheduled for outpatient follow-up. A transthoracic echocardiogram was performed a week later, which demonstrated an ejection fraction of 50% and a normal functioning tricuspid valve bioprosthesis.

**Table 1:** Antibiogram demonstrating organisms and susceptibilities

Blood Culture Organisms	Sensitive to
Pseudomonas aeruginosa	Gentamicin, Cefepime, Piperacillin/Tazobactam, Meropenem
Gemella hemolysans	Ampicillin, Clindamycin, Piperacillin/Tazobactam, Vancomycin
Streptococcus anginosus	Ampicillin, Cefepime, Piperacillin/Tazobactam, Vancomycin

## Discussion

This previously healthy 30-year-old female had polymicrobial right-sided endocarditis that resulted in third-degree AV block (complete heart block), septic pulmonary emboli, acute hypoxic respiratory failure, and heart failure with reduced ejection fraction (HFrEF). IE is often caused by *Streptococcus viridans* or *S. aureus*. The endocarditis described in our case report is unique due to the severity of the disease and the microorganisms that were isolated on cultures: *P. aeruginosa*, *G. hemolysans*, and *S. anginosus*. 

*P. aeruginosa* is a gram-negative, aerobic, bacillus. Bacteremia from *P. aeruginosa* is often hospital-acquired acquired while endocarditis is uncommon; it is often associated with IV drug use and prosthetic heart valves. *P. aeruginosa* is the culprit microorganism in approximately 3% of patients with IE [2]. *P. aeruginosa* endocarditis has been associated with high morbidity and mortality as well as antibiotic resistance. It has been associated with a mortality rate of 80% [3] and approximately 56% of *P. aeruginosa* cases require surgery [2]. Complications include heart failure and arterial emboli [2]. Our patient demonstrated evidence of both heart failure and emboli, which has been often observed with this type of endocarditis. Reviews have demonstrated rates of relapse as high as 33% and these patients had significantly higher mortality. Combination therapy with two intravenous antipseudomonal antibiotics from different classes to which the microbe is vulnerable is suggested with a therapy duration of six weeks; an aminoglycoside antibiotic is frequently chosen unless the use is precluded by nephrotoxicity [4]. Nevertheless, no clinical evidence has been documented that the usage of two antipseudomonal antibiotics offers a decrease in mortality rate [4]. *P. aeruginosa* infections are complicated by biofilm formation and by the possible emergence of antibiotic resistance during treatment due to genetic modifications such as alterations in efflux pumps and reductions in porin expression [4].

 Whereas *G. hemolysans* is a gram-positive coccus and it has been found in the oropharynx, the genitourinary system, and the gastrointestinal system [5]. *G. hemolysans* endocarditis has been rarely observed. IE due to *G. hemolysans* has been seen in patients with previously damaged valves, those with poor dental states, and those with GI malignancies [5]. Literature indicates that the production of exopolysaccharide may contribute to its capacity to cause endocarditis and that nearly 50% of *Gemella *endocarditis cases require surgery [5]. Gemella species have been found to be vulnerable to β-lactams and vancomycin. A combination of penicillin G and gentamicin is the current treatment of choice for these infections, whereas vancomycin can be used in patients with penicillin allergy [5]. Our patient had bacteremia with this pathogen that resolved with antibiotic treatment and ultimately required surgical intervention. There was no evidence to conclude our patient had proper dental care; therefore, poor dental status remains a possible risk factor. There was no PMH suggesting she had a previously damaged valve but due to her risk factors, it is possible she may have had prior damage to her valves. A recent review of the literature revealed that there have been 24 documented cases of *G. hemolysans* endocarditis [6], with ours being the 25th and to the best of our knowledge the only one causing significant tricuspid valve destruction. 

*S. anginosus* belongs to the *Streptococcus milleri* group. Unlike *S. viridans*, it is rarely a cause of endocarditis. It frequently colonizes the oropharynx, the GI tract, and the genitourinary tract [7]. These infections tend to be invasive and pyogenic [7]. *S. milleri* group endocarditis has higher rates of pseudoaneurysms, intracardiac abscesses, and central nervous system emboli [8].

Our patient had polymicrobial endocarditis with these unusual and difficult to treat organisms. Multi-pathogen endocarditis is rare. More than 90% of IE cases are caused by a single pathogen. The incidence of polymicrobial infection ranges from 1% to 6% in the general population and is slightly higher in those with risk factors [9]. Previous research has determined that a single organism can predispose and create a niche for other microorganisms to inhabit [9]. However, extensive research describing single organisms leading to polymicrobial endocarditis has not been performed. The most common dual pathogen combination in previous studies was coagulase-negative *Staphylococci *with enterococci [9]. Polymicrobial infections have been reported to have poor outcomes, but differences between multi-organism versus single organism IE have not been sufficiently studied to determine if polymicrobial endocarditis infections have worse overall outcomes [9].

As per the American Association for Thoracic Surgery guidelines, surgery should be considered in patients with right-sided IE when large vegetations (>20 mm) are present and the patient has persistent bacteremia or fevers lasting longer than five to seven days after initiation of appropriate antimicrobial therapy, or in those with evidence of septic pulmonary embolism [10]. Our patient met these criteria and necessitated surgery for the control of her infection.

## Conclusions

This patient was young and immunocompetent but had the risk factor of being an IV drug user. By the time she presented to the hospital, she was critically ill, her valve had deteriorated, and surgery was inevitable. Perhaps, the reason for the complete destruction of her valve was the pathogenicity of the organisms involved. Their virulence factors, and invasiveness, compounded by their combined infection resulted in the complete annihilation of her tricuspid valve. Although polymicrobial endocarditis has poor outcomes, a week after the operation, her ejection fraction had improved to 50% and she only exhibited mild tricuspid valve regurgitation. Six weeks later, she was in a stable condition and presented for follow-up. Surgery is necessitated in nearly 50% of *Gemella *endocarditis cases, 62% of cases with *S. anginosus* group, and approximately 56% of *P. aeruginosa* cases. To our knowledge, this is the only case of polymicrobial endocarditis with these three pathogens simultaneously occurring in a young, immunocompetent patient.
